# Non-invasive markers of cardiovascular risk in patients with subclinical hypothyroidism: A systematic review and meta-analysis of 27 case control studies

**DOI:** 10.1038/s41598-018-22897-3

**Published:** 2018-03-15

**Authors:** Kecheng Yao, Tianming Zhao, Linghai Zeng, Jianming Yang, Yanqun Liu, Qian He, Xiulan Zou

**Affiliations:** 1 Department of Gerontology, The People’s Hospital of China Three Gorges University & The First People’s Hospital of Yichang, Yichang, 443000 Hubei Province China; 2 Department of Respiratory and Critical Care Medicine, The People’s Hospital of China Three Gorges University & The First People’s Hospital of Yichang, Yichang, 443000 Hubei Province China; 3 Department of Endocrinology, The People’s Hospital of China Three Gorges University & The First People’s Hospital of Yichang, Yichang, 443000 Hubei Province China

## Abstract

It has been reported that subclinical hypothyroidism (SCH) is closely related to subclinical atherosclerosis. According to the impact of SCH on noninvasive markers of cardiovascular risk, we fulfilled a meta-analysis of included studies to provide an integrated overview. We searched electronic databases and included all relevant studies involving SCH and epicardial adipose tissue (EAT), carotid intima-media thickness (CIMT), pulse wave velocity (PWV), flow-mediated dilation (FMD) and glyceryl trinitrate-induced dilation (GNT- induced dilation). The result was calculated in a meta-analysis to assess the impact of SCH on these markers. A total of 27 studies were entered in the final analysis. Compared with euthyroid subjects, SCH patients exhibited a significantly increased CIMT (SMD: 0.369 mm; 95%CI: 0.038, 0.700; P = 0.029) and EAT (SMD: 1.167 mm; 95%CI: 0.869, 1.466; P = 0.000) and increased PWV (SMD: 3.574 m/s; 95%CI: 0.935, 6.213, P = 0.008). We also found significantly lower FMD (SMD: −1.525%, 95%CI: −2.156, −0.894, P = 0.000) and lower GNT-induced dilation (SMD: −0.384%, 95%CI: −0.625, −0.142, P = 0.002). Sensitivity analysis and subgroup analysis confirmed the above results. Our meta-analysis confirmed a significant association of SCH and cardiovascular risk with arterial wall thickening and stiffening and endothelial dysfunction. These findings will help to establish detailed cardiovascular prevention strategies for SCH patients.

## Introduction

Subclinical hypothyroidism (SCH) is a disease characterized by the absence of distinct clinical symptoms and signs; laboratory examination of patients with SCH have revealed elevated serum levels of thyroid-stimulating hormone (TSH) with normal serum free thyroxine concentrations^1^. According to the level of TSH, SCH can be divided into two types: TSH levels less than 10 mIU/L (normal thyroxine) is considered mild SCH, whereas TSH levels greater than or equal to 10 mIU/L is considered severe SCH^2,3^. The United States National Health and Nutrition Survey noted that among the population >12 years of age, considering 4.5 mIU/L as the upper limit of normal TSH levels corresponded to a SCH prevalence rate of 4.3%^4^. According to the Colorado survey, considering 5.0 mIU/L as the upper limit of normal TSH levels corresponded to a SCH prevalence of 8.5%^1^.

Most patients with SCH do not exhibit clinical symptoms, but SCH can exert a detrimental effect on the cardiovascular (CV) system. The major adverse effects of SCH include reduce cardiac diastolic function and accelerated atherosclerosis^5^, impaired endothelial dysfunction^6^, increased carotid artery intima-media thickness (CIMT)^7^, induced coronary artery disease and increased mortality of coronary heart disease^8,9^. Studies have shown that abnormal lipid metabolism and oxidative stress are involved in the occurrence of these adverse effects^7,10^. To further understand the adverse effects induced by SCH, a growing number of studies have focused on CV with disease-related indicators to evaluate the correlation between SCH and atherosclerosis.

CIMT can be measured by noninvasive ultrasound imaging to evaluate subclinical atherosclerosis^11,12^. CIMT has been widely accepted as a formidable predictor of adverse CV disease events (shock and myocardial infarction)^13,14^. Similarly, flow-mediated dilation (FMD), pulse wave velocity (PWV), and glyceryl trinitrate-induced dilation (GNT-induced dilation) as independent predictors of CV events are considered to be surrogate markers of subclinical atherosclerosis^15–18^. FMD and GNT-induced dilation are widely used to detect vascular endothelial function as a noninvasive and accurate detection technique^19^. In addition, PWV has been used to detect peripheral and central arterial stiffness^20^. The epicardial adipose tissue (EAT) thickness surveyed by echocardiography is also considered an important independent predictor of an increased incidence of CV events for evaluating subclinical coronary atherosclerosis^21^.

For the past few years, many case-control studies have announced that SCH patients exhibit accelerated atherosclerosis, damaged endothelial function, and increased arterial stiffness. However, small sample sizes and potential confounding factors influence the strength of previous evidence. According to the impact of SCH on noninvasive markers of CV risk, we fulfilled a systematic review and meta-analysis of included studies to provide an integrated overview.

## Methods

We strictly followed the PRISMA reporting specifications for this meta-analysis.

### Search strategy

According to the PRISMA guidelines^22^, we formulated a detailed search strategy, including SCH and noninvasive markers of CV risk (i.e., IMT, EAT, PWV, FMD, and GNT-induced dilation). We systematically searched online databases (PubMed, Embase, Web of Science) through 30 November 2017. All possible combinations of search terms included (‘Subclinical hypothyroidism’) AND (‘atherosclerosis’) AND (‘arterial stiffness’) AND (epicardial adipose tissue OR carotid intima-media thickness OR pulse wave velocity OR flow-mediated dilation OR glyceryltrinitrate-induced dilation). No language or publication date restrictions were imposed in this study.

Furthermore, manual audits were conducted on all retrieved articles. If data were missing from an article, the authors communicated via e-mail to try to obtain the raw data. Two independent authors (YAO K and ZOU X) analysed each paper and independently extracted the data. In the case of differences in opinion, the two authors consulted with a third investigator (ZHAO T). The differences were settled by accordance. The selected results were determined by unanimous agreement of each author and were reported according to the PRISMA guidelines.

### Inclusion criteria

Studies adhering to the following inclusion criteria were included: 1) case-control studies; 2) including subjects with normal thyroid function as a control group, 3) CIMT, EAT, PWV, FMD and GNT-induced dilation indicators of patients with SCH compared with euthyroid (EU) subjects reported; and 4) 95% confidence intervals (CIs) were reported.

### Exclusion criteria

During the literature screening process, studies characterized by the following criteria were excluded: 1) including participants diagnosed with severe SCH or hyperthyroidism; 2) including participants being treated with medicine; 3) including patients with abnormal thyroid function who were diagnosed thyroid disease; 4) studies without a control group, animal studies, and reviews; 5) studies that did not provide the value of at least one variable (mean and standard deviation) among the predictors.

### Data extraction and quality assessment

For each included study, we extracted the data corresponding to the demographic variables, the number of cases and controls, and the major clinical variables including CIMT, FMD, GNT-induced dilation, PWV and EAT.

Next, the methodological quality of each study was evaluated based on the characteristics of the study. The Newcastle Ottawa scale (NOS) was used to assess the quality of nonrandomized observational studies^23^. The scoring system consisted of three major areas (selection, comparability, exposure), and the results ranged from 0 to 8 with higher scores representing better methodological quality. The quality evaluation results of the NOS are reported in Table 1.

**Table 1:** Characteristics of included studies.

Author	Year	Country	Participants (SCH/EU)	Age (SCH/EU)	Study design	TSH cutoff value	T4 measured?	Measure outcomes	NOS Score
Ali Aydogdu^33^	2017	Turkey	30/30	37.70 ± 13.54/40.96 ± 13.39	Case-control study	≥5.5 mIU/L	+	EAT thickness	7
Pramila Kalra^39^	2016	Canada	58/49	31.83 ± 8.91/32.42 ± 9.98	Case-control study	>4.5 mIU/L	+	PWV	5
Edip Unal^50^	2017	Turkey	38/38	8.1 ± 3.6/8.9 ± 2.4	Case-control study	>4.2 mIU/L	+	CIMT	8
Irmak Sayin^48^	2016	Turkey	44/42	41.2 ± 15.9/42.1 ± 13.5	Case-control study	>5.0 mIU/L	+	EAT thickness	8
Mustafa Altay^30^	2017	Turkey	35/30	34.4 ± 10.3/32.5 ± 7.5	Case-control study	—	+	CIMT	4
Yasemin Isik-Balci^38^	2016	Turkey	53/31	9.25 ± 4.29/7.19 ± 5.15	Case-control study	—	+	CIMT	4
Dilek Arpaci^31^	2016	Turkey	41/35	34.07 ± 6.70/31.82 ± 5.57	Case-control study	>5.4 mIU/L	+	EAT thickness	7
Nasmi Niknam^47^	2016	Iran	25/25	35.9 ± 7.6/37.5 ± 7.3	Case-control study	>4.2 mIU/L	+	CIMT FMD	7
Manuela Cerbone^35^	2016	Italy	31/31	9.18 ± 3.56/9.45 ± 3.62	Case-control study	>4.5 mIU/L	+	CIMT FMD	8
Erdal Belen^34^	2015	Turkey	51/51	48.6 ± 8.5/49.1 ± 7.9	Case-control study	>4.8 mIU/L	+	EAT thickness	7
Gulhan Akbaba^28^	2016	Turkey	51/43	36.9 ± 10.6/34.9 ± 8.4	Case-control study	>4.0 mIU/L	+	CIMT	8
Mustafa Unubol^51^	2014	Turkey	37/25	40.08 ± 11.62/38 ± 12.71	Case-control study	>4.0 mIU/L	+	EAT thickness	7
Ismail Dogu Kilic^41^	2013	Turkey	32/29	41.5 ± 12.0/38.1 ± 11.4	Case-control study	>4.2 mIU/L	+	CIMT FMD GTN-induced dilatation	5
Mehmet Asik^32^	2013	Turkey	33/32	38.18 ± 15.06/39.41/9.74	Case-control study	>5.49 mIU/L	+	CIMT EAT thickness	7
Levent Korkmaz^43^	2013	Turkey	61/24	44 ± 14/43 ± 17	Case-control study	>4.94 mIU/L	+	EAT thickness	7
Guangda Xiang^54^	2012	China	10/10	34.2 ± 5.8/34.6 ± 5.3	Case-control study	>5.5 mIU/L	+	FMD GTN-induced dilatation	7
Velkoska Nakova Valentina^52^	2011	Macedonia	67/30	42.4 ± 16.2/43.6 ± 12.8	Case-control study	>4.2 mIU/L	+	CIMT	8
Esat Erdem Türemen^49^	2011	Turkey	37/23	46.35 ± 11.41/42.61 ± 11.61	Case-control study	—	+	FMD GTN-induced dilatation	6
Levent Kebapcilar^40^	2010	Turkey	38/19	49.79 ± 10.04/49.95 ± 8.12	Case-control study	>5.0 mIU/L	+	CIMT	8
Xiang GD^37^	2010	China	40/18	57 ± 9/56 ± 8	Case-control study	>5.5 mIU/L	+	FMD GTN-induced dilatation	8
SOO-KYUNG KIM^42^	2009	Korea	36/32	36.0 ± 6.2/36.1/5.4	Case-control study	>5.5 mIU/L	+	CIMT	8
Guangda Xiang^53^	2009	China	30/27	53.0 ± 8/52.0 ± 7	Case-control study	>5.5 mIU/L	+	CIMT	8
Toshiki Nagasaki(a)^45^	2007	Japan	42/42	66.0 ± 2.6/64.7 ± 3.2	Case-control study	>3.8 mIU/L	+	PWV	8
Toshiki Nagasaki(b)^46^	2007	Japan	40/50	63.2 ± 2.7/64.3 ± 3.1	Case-control study	>3.8 mIU/L	+	PWV	8
CARLA A. DE ALMEIDA^29^	2007	Brazil	30/27	43.07 ± 9.76/43.19 ± 8.39	Case-control study	>4.0 mIU/L	+	CIMT	8
Toshiki Nagasaki^44^	2006	Japan	50/50	65.2 ± 2.6/64.3 ± 3.1	Case-control study	>4.7 mIU/L	+	PWV	8
Ayse S. Cikim^36^	2004	Turkey	25/23	32.28 ± 9.67/35.87 ± 7.93	Case-control study	>4.2 mIU/L	+	CIMT FMD	8

### Statistical analysis and risk of bias assessment

Statistical analysis was performed by STATA 12.0 software. Differences among cases and controls were presented as the standardized mean difference (SMD) with the associated 95%CI for continuous variables^24^.

All reported P values were two-sided with a significance level set at P < 0.05. Heterogeneity between studies was calculated by I^2^ statistics; an I^2^ value of 0% indicates no heterogeneity, and I^2^ values of 25%, 25–50%, and 50% indicate low, moderate, and high heterogeneity, respectively^25^.

Begg’s test, Egger’s test and funnel plots were used to test publication bias. We visually examined the symmetry of the funnel plots to evaluate possible small sample effects, and we used Begg’s test and Egger’s test to evaluate publication bias of the included studies. Statistical significance was considered for P < 0.10^26^. When publication bias occurred, the adjusted effect scale was assessed using the Duval and Tweedie’s trim and fill method with the random-effect model^27^.

Considering the variability among the studies, we used a random-effect method for all analyses.

### Sensitivity analyses

Sensitivity analysis is used to investigate the reliability of a meta-analysis. We evaluated the reliability of the conclusions by examining the impact of individual studies on the total merged effect. For each study, a new meta-analysis was performed upon deleting that study to assess the stability of the results compared with the total effect.

### Subgroup analysis

Taking into account the potential impact of SCH confounding factors on the outcomes of the study, we conducted subgroup analyses for smoking, obesity, NOS score, and national factors.

### Meta-regression analyses

Meta-regression is often used to explore the sources and sizes of heterogeneity among studies and to further explain the effects of heterogeneity in the meta-analysis. We hypothesized that the included studies could exhibit differences in the demographic variability (sample size) and the combined traditional CV risk factors (smoking habit, diabetes mellitus, obesity, hyperlipidaemia and hypertension). To assess the possible effects of these variables in explaining different results observed across studies, a regression model with changes in CIMT, FMD, PWV and EAT values as dependent variables (y) and the abovementioned co-variates as independent variables (x) was constructed.

### Availability of data and materials

All the data we get was from public sources.

## Results

After excluding the repeated citations, we retrieved 423 articles. Among these studies, 217 were excluded because they deviated from the theme according to the title and/or abstract, 15 because they were animal/non-case-control studies or because they lacked the data of interest.

**Figure 1:**
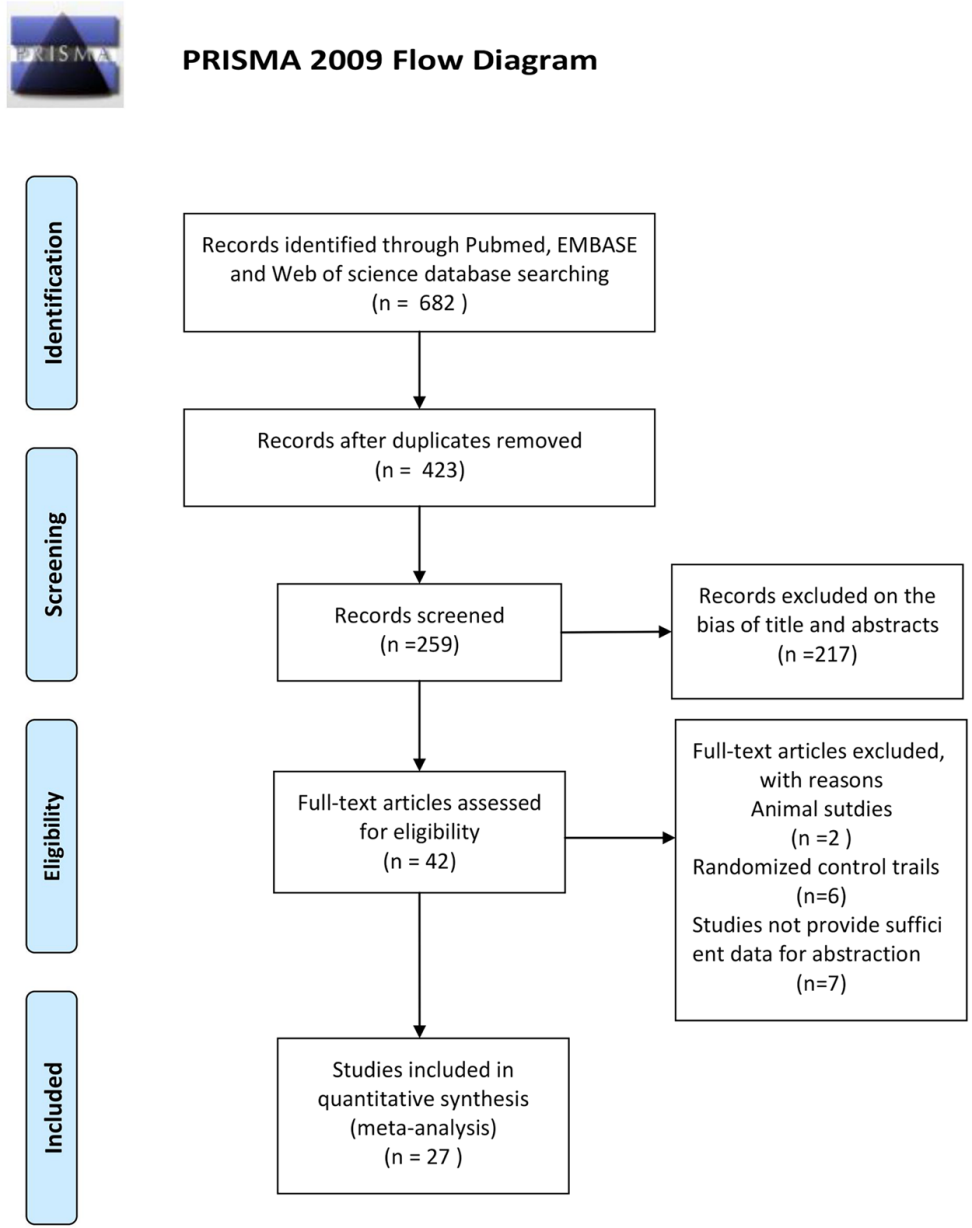
PRISMA 2009 flow diagram.

Thus, 27 studies (on 1065 SCH cases and 866 subjects) were included in the final analysis^28–54^ (Fig. 1). Thirteen studies compared the CIMT between 494 SCH patients and 390 EU subjects. PWV was evaluated in 5 studies (248 cases and 240 controls), 9 studies reported FMD (230 patients and 204 controls), 7 studies reported EAT (297 cases and 239 controls), and 6 studies reported GNT-induced dilation (149 cases and 125 controls).

### Study characteristics

All the included studies were case-control designs. The main characteristics of the studies are shown in Table 1.

The number of patients varied from 10 to 67, the mean age ranged from 8.1 to 65.2 years. Smoking habit was reported by 4 studies^28,36,41,52^, obesity by 4 studies^28,32,40,52^, hypertensive by only 1 study^36^ and hyperlipidaemia by 4 studies^30,41,42,52^.

The NOS for quality assessment of the included studies yielded a median value of 6. There were 23 high-quality articles in our meta-analysis^28,29,31–37,40,42–54^.

### Subclinical hypothyroidism versus euthyroidism

**Figure 2:**
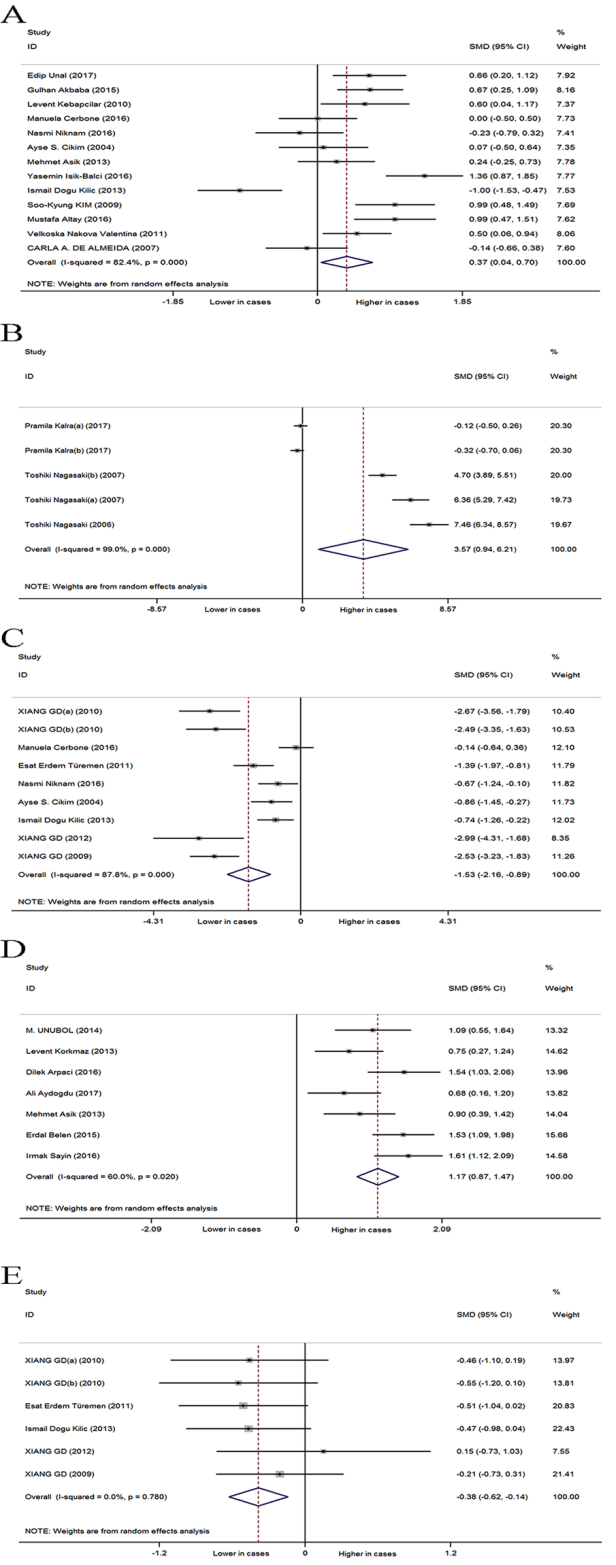
Forest plots for the effect of SCH patients on cardiovascular risk markers including carotid intima-media thickness (**A**), pulse wave velocity (**B**), flow-mediated dilation (**C**), epicardial adipose tissue (**D**) and glyceryl trinitrate-induced dilation (**E**).

Analysis of 13 studies, revealed a significantly higher CIMT in 494 SCH patients than in 390 EU subjects (SMD: 0.369 mm; 95%CI: 0.038, 0.700; P = 0.029, Fig. 2A), with significant heterogeneity among studies (I^2^ = 82.4%; P = 0.000).

Analysis of 5 studies, evaluating a total of 248 cases and 240 controls, indicated that SCH patients have a significantly higher PWV than EU subjects (SMD: 3.574 m/s; 95%CI: 0.935, 6.213, P = 0.008, Fig. 2B), and the heterogeneity among studies was significant (I^2^ = 99%, P = 0.000).

Nine studies, evaluating a total of 230 SCH patients and 204 controls, indicated that SCH patients have a significantly lower FMD than EU subjects (SMD: −1.525%, 95%CI: −2.156, −0.894, P = 0.000, Fig. 2C), with significant heterogeneity among studies (I^2^ = 87.8%; P = 0.000).

Seven studies, evaluating a total of 297 SCH patients and 239 EU subjects, revealed a significantly higher EAT in SCH patients than in EU subjects (SMD: 1.167 mm; 95%CI: 0.869, 1.466; P = 0.000, Fig. 2D), with significant heterogeneity among studies (I^2^ = 60%; P = 0.02).

Six studies, evaluating a total of 149 SCH patients and 125 controls, showed that SCH patients have a significantly lower GNT-induced dilation than EU subjects (SMD: −0.384%, 95%CI: -0.625, −0.142, P = 0.002, Fig. 2E), without heterogeneity among studies (I^2^ = 0%; P = 0.78).

**Figure 3:**
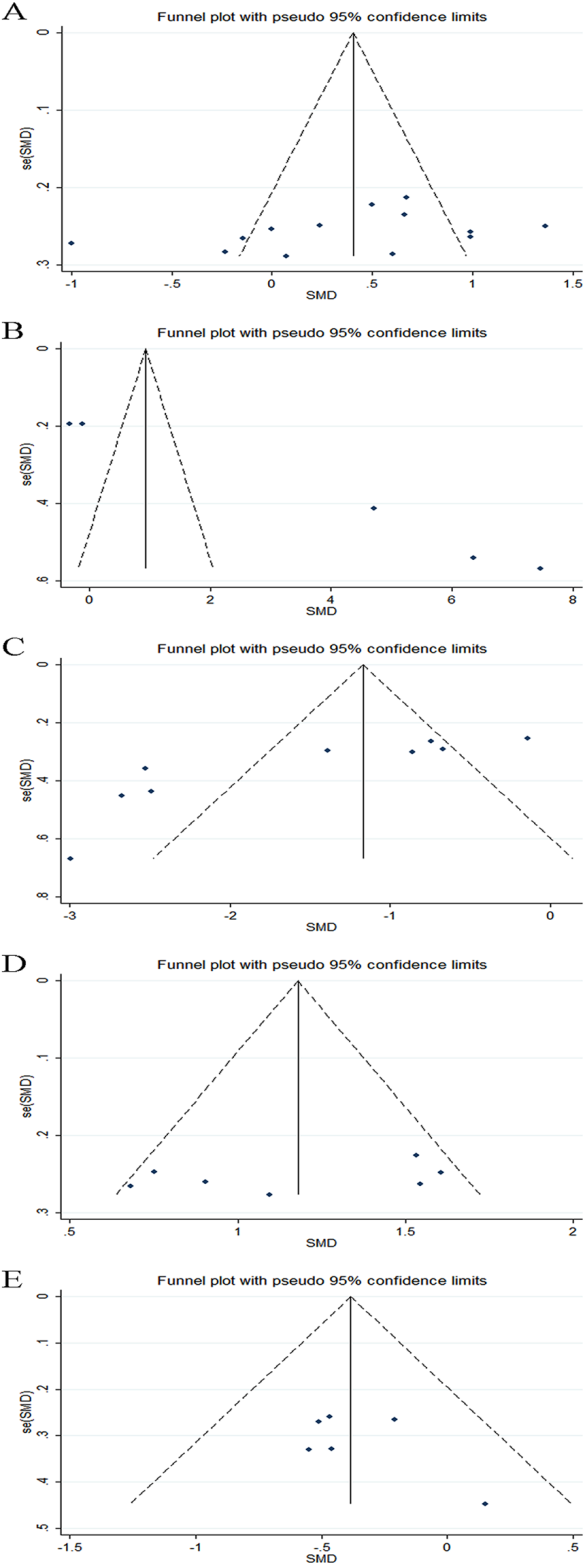
Visual evaluation of the funnel plot for the included studies on SCH patients and EU subjects: carotid intima-media thickness (**A**), pulse wave velocity (**B**), flow-mediated dilation (**C**), epicardial adipose tissue (**D**) and glyceryl trinitrate-induced dilation (**E**).

**Figure 4:**
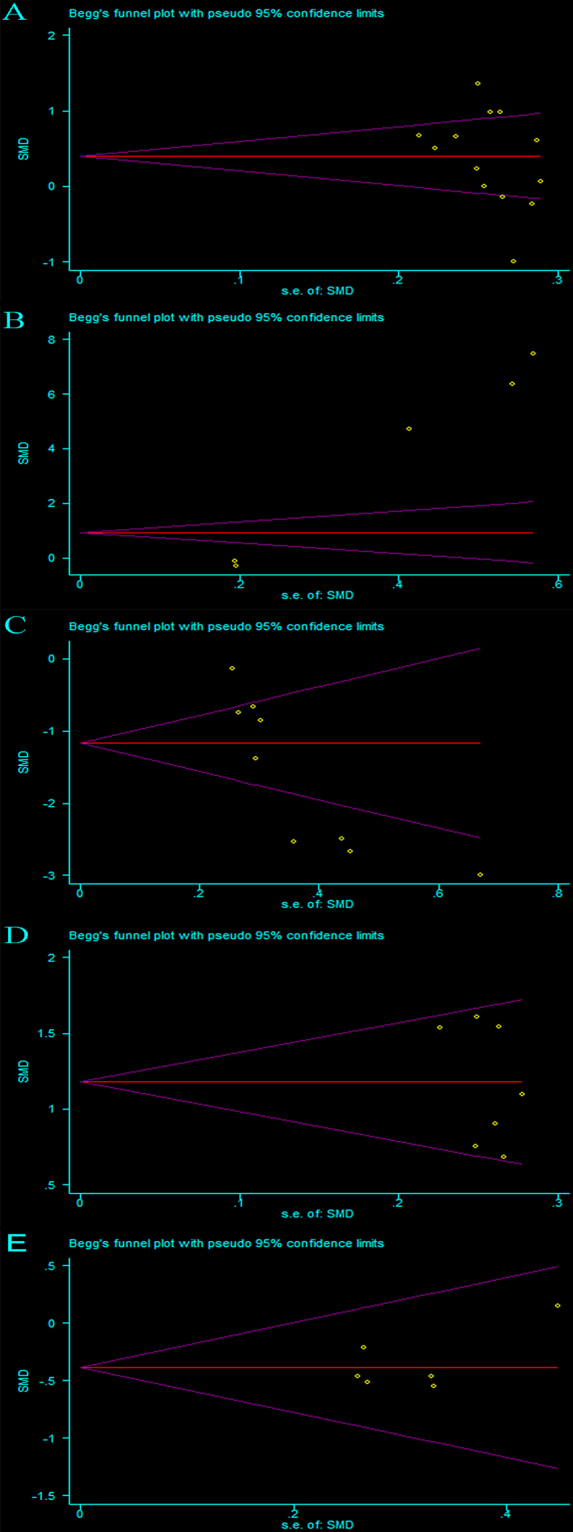
Begg’s test for publication bias on including studies: carotid intima-media thickness (**A**), pulse wave velocity (**B**), flow-mediated dilation (**C**), epicardial adipose tissue (**D**) and glyceryl trinitrate-induced dilation (**E**).

Publication bias in included studies may affect the results of meta-analyses. Therefore, we used funnel plots to assess potential publication bias among our included studies.Visual evaluation of the funnel plot for the included studies on SCH patients and EU subjects indicated low publication bias for CIMT (Fig. 3A), confirmed by Begg’s test (P = 0.127, Fig. 4A) and Egger’s test (P = 0.196). Similarly, no publication bias was found for EAT and GNT-induced dilation by inspection of the funnel plots (Fig. 3D,E), confirmed by Begg’s test (P = 0.548, Fig. 4D; P = 1.0, Fig. 4E, respectively) and Egger’s test (P = 0.328, P = 0.291, respectively). In comparison, an asymmetric distribution of studies was detected among those that assessed PWV (Fig. 3B) and FMD (Fig. 3C), and Begg’s test (P = 0.086, Fig. 4B; P = 0.029, Fig. 4C, respectively) and Egger’s test (P = 0.000, P = 0.004) confirmed significant publication bias.

We performed sensitivity analysis by deleting single studies one-by-one and performing an additional meta-analysis for each study removed. For CIMT, PWV, FMD, EAT and GNT-induced dilation, the influence of the omission of each individual study on pooled SMD was assessed. However, no single study had an impact on the overall effect according to the sensitivity analysis, and thus, the meta-analysis was statistically stable.

To eliminate the effects of confounding factors on the outcomes of the study, we conducted a subgroup analysis for high-heterogeneity markers. Subgroup analysis using the high-quality studies (n = 10) indicated that CIMT was significantly increased in patients with SCH (SMD: 0.35 mm; 95%CI: 0.10, 0.59; P = 0.005), and the heterogeneity of the result was markedly reduced (I^2^ = 59%; P = 0.009). For PWV, subgroup analysis revealed that smoking habit (n = 3) was significantly increased in patients with SCH (SMD: 6.138 m/s; 95%CI: 4.493, 7.783; P = 0.005), with significant heterogeneity among studies (I^2^ = 87.9%; P = 0.00). For FMD, we assessed the variability of heterogeneity and effects by region classification, and we found that the heterogeneity of China studies (n = 3) (SMD: −2.607%; 95%CI: −3.044, −2.170; P = 0.000) (I^2^ = 0%; P = 0.925) and other national studies (n = 5) (SMD: −0.745%; 95%CI: −1.146, −0.344; P = 0.000) (I^2^ = 62.3%; P = 0.031) was significantly decreased; the pooled effects were statistically significant, and the heterogeneity of the results was significantly decreased. For EAT, we assessed the variability of heterogeneity and effects of non-obesity (n = 2), and we found that SCH was increased (SMD: 1.538 mm; 95%CI: 1.202, 1.874; P = 0.000) without heterogeneity among studies (I^2^ = 0%; P = 0.978).

**Figure 5:**
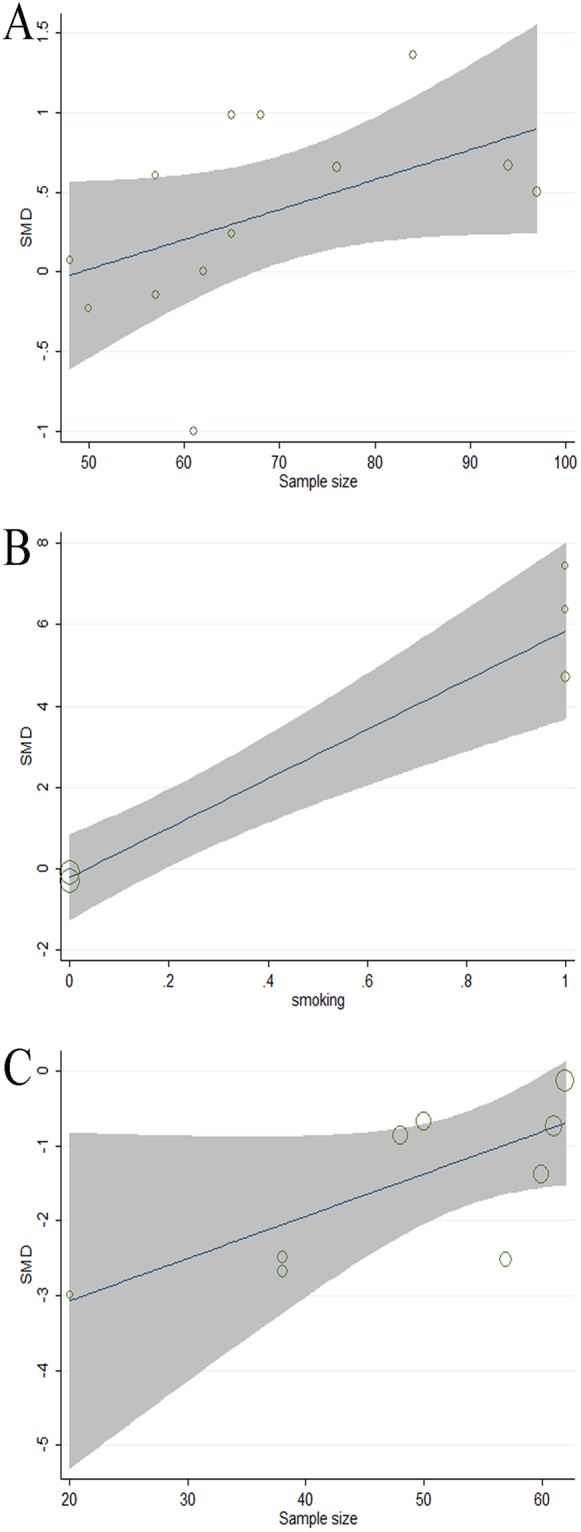
Meta-regression analyses: impact of demographic and clinical variables on effect size assessed with regression techniques. Subclinical hypothyroidism versus euthyroidism: effect of sample size on carotid intima-media thickness (**A**), smoking habit on pulse wave velocity (**B**), and sample size on flow-mediated dilation (**C**).

Regression models for studies comparing SCH patients and EU subjects revealed that small-size studies significantly affected CIMT, indicating that small-sample-size studies are the source of heterogeneity (p = 0.075, tau^2^ = 0.2467, Adj R-squared = 23.39%, I-squared_res = 78.41%, Fig. 5A). Similarly, a small sample size was also associated with FMD heterogeneity (p = 0.041, tau^2^ = 0.5246, Adj R-squared = 44.21%, I-squared_res = 82.69%, Fig. 5C).According to the regression models for studies of SCH patients and EU subjects, smoking habit (p < 0.01, tau^2^ = 0.9931, Adj R-squared = 92.41%, I-squared_res = 82.44%, Fig. 5B) as a confounding factor significantly affected PWV. No other demographic or clinical factors influenced the assessment results.

## Discussion

This meta-analysis encompassing 27 studies and including 1065 SCH patients and 866 EU subjects provides compelling evidence that SCH was associated with increased arterial stiffness. We also found a marked increase in EAT and impaired endothelial function (as expressed by a reduced FMD and GNT-induced dilation) in SCH patients compared with EU controls. The sensitivity analysis results strongly confirmed our findings. In addition, the regression models provide evidence that smoking habits and small sample sizes have significant effects on the results of assessments. A former published meta-analysis reported an increase in CIMT in patients with SCH^55^. Our meta-analysis confirms this result and further reveals significant adverse effects of PWV, EAT, FMD and GNT-induced dilation as other CV risk factors.

There is frequent concern regarding the long-term adverse effects of various factors on increasing the risk of CV disease in SCH patients. SCH is associated with increased levels of total cholesterol and low-density lipoprotein cholesterol. In a population-based study of the Health Aging and Body Composition, the authors prospectively investigated the adverse effects of TSH levels on CV outcomes through a 4-year follow-up. The study found that when the patient’s TSH levels exceeded 7 mIU/L and 10 mIU/L, the risk ratios for developing heart failure were 2.58 and 3.26, respectively^56^. Another study conducted by Rodondi *et al*. revealed that more than 55,000 individuals exhibited a positive association of elevated TSH levels with CV events rates and mortality^8^.

Many CV risk factors are deemed to play causal roles in the atherosclerotic process^57^. SCH contributes to vascular endothelial dysfunction by promoting lipid metabolism disorder. A study carried out in DaDong district of Shenyang city (China) revealed that the elevated serum TSH levels are positively correlated with serum total cholesterol, triglyceride, and low-density lipoprotein cholesterol and negatively correlated with high-density lipoprotein cholesterol^58^. Although the majority of SCH patients are hyperlipidaemic, the correlation between subclinical atherosclerosis and SCH appear to be more complicated, and dyslipidaemia may not fully explain the accelerating atherosclerosis in this clinical setting^10,59^. There is increasing evidence that long-term exposure to high levels of TSH has a deleterious effect on the CV system beyond the mechanism of dyslipidaemia^9,33,49,60–62^. In addition, chronic inflammation may initiate and promote atherosclerosis and its complications through adverse effects on the vascular endothelium, which may be one of the causes of endothelial dysfunction. The clinical importance of SCH in CV disease and mortality remains controversial, and many studies offer conflicting results. This can be explained by the choice of heterogeneous patient groups, arbitrary definitions of TSH reference limits when defining SCH, lack of stratification based on elevated TSH levels, and different study designs.

To fully understand the correlation between SCH and subclinical atherosclerosis, our meta-analysis involves major CV risk markers. In addition, to prevent SCH-related dyslipidaemia from affecting the results of our assessment, we compared hyperlipidaemic and non-dyslipidaemic SCH patients, and the results were consistent. In addition, a meta-regression analysis was conducted to assess whether clinical data and demographic variables affect the outcome. As expected, the regression models revealed that the combination of traditional CV risk factors (e.g., smoking habits, hyperlipidaemia) and small-sample-size studies affect the outcomes. In addition, FMD and GNT-induced dilation were significantly impaired and PWV was significantly increased in SCH patients compared with EU subjects. However, given the small number of studies included in the PWV results, these results should be interpreted with caution. Studies have shown that when CIMT increases by 0.163 mm, the risk of myocardial infarction increases by 43%^63,64^; furthermore, the risk of CV events increases by 14% when PWV increases by 1 m/s^16^. These data illustrate the clinical significance of our assessment of these indicators and the need for regular examination of the relevant indicators of subclinical atherosclerosis in patients with SCH.

Some potential limitations of this study need to be considered. First, among the studies included in this meta-analysis, each study had their own inclusion and exclusion criteria. Some of these patients were characterized by CV risk factors (smoking, obesity, diabetes mellitus, hyperlipidaemia and hypertension). Although the results of the regression analysis could be improved by assessing the impact of most clinical and demographic variables on the outcomes of the observation, caution is needed in interpreting the overall outcome. Finally, the evaluation of heterogeneity among studies is very important. Although all sources of possible heterogeneity could not be conclusively determined, the stability of the outcomes was confirmed after adjusting for potential publication bias.

In conclusion, SCH has a significant association with arterial wall thickening and stiffening and endothelial dysfunction and increased risk of CV events. Therefore, Whether SCH patients can benefit from early assessments of measures of CV risk markers that may require large-scale, long-term clinical studies to further confirmation.
